# Prevention of gastrointestinal lead poisoning using recombinant *Lactococcus lactis* expressing human metallothionein-I fusion protein

**DOI:** 10.1038/srep23716

**Published:** 2016-04-05

**Authors:** Xue Xiao, Changbin Zhang, Dajun Liu, Weibin Bai, Qihao Zhang, Qi Xiang, Yadong Huang, Zhijian Su

**Affiliations:** 1 Department of Cell Biology, College of Life Science and Technology, Jinan University, Guangzhou 510632, P.R. China; 2 Guangdong Provincial Key Laboratory of Bioengineering Medicine, Institute of Biomedicine, Jinan University, Guangzhou 510632, P.R. China; 3 Department of Clinical Molecular Diagnosis, Guangdong Women and Children Hospital, Guangzhou 510010, P.R. China; 4 Department of Food Science and Engieering, Jinan University, Guangzhou, 510632, P.R. China

## Abstract

Low-level lead poisoning is an insidious disease that affects millions of children worldwide, leading to biochemical and neurological dysfunctions. Blocking lead uptake via the gastrointestinal tract is an important prevention strategy. With this in mind, we constructed the recombinant *Lactococcus lactis* strain pGSMT/MG1363, which constitutively expressed the fusion protein glutathione S-transferase (GST)–small molecule ubiquitin-like modifier protein (SUMO)–metallothionein-I (GST-SUMO-MT). The thermodynamic data indicated that the average number of lead bound to a GST-SUMO-MT molecule was 3.655 and this binding reaction was a spontaneous, exothermic and entropy-increasing process. The total lead-binding capacity of pGSMT/MG1363 was 4.11 ± 0.15 mg/g dry mass. Oral administration of pGSMT/MG1363 (1 × 10^10^ Colony-Forming Units) to pubertal male rats that were also treated with 5 mg/kg of lead acetate daily significantly inhibited the increase of blood lead levels, the impairment of hepatic function and the decrease of testosterone concentration in the serum, which were all impaired in rats treated by lead acetate alone. Moreover, the administration of pGSMT/MG1363 for 6 weeks did not affect the serum concentration of calcium, magnesium, potassium or sodium ions. This study provides a convenient and economical biomaterial for preventing lead poisoning via the digestive tract.

Lead poisoning has been recognized as one of the most important environmental diseases, affecting millions of people worldwide^1^,^2^. Leaded gasoline, lead-based paint, traditional medicines, electronics industrial emissions and contaminated foods and water are the main sources of lead exposure^3^,^4^. Lead can cause adverse effects that include neurotoxicity, nephrotoxicity, reproductive toxicity and deleterious effects on the immune, hematological and cardiovascular systems^5^,^6^,^7^,^8^. These mechanisms of toxicity are related to lead’s ability to inhibit or mimic the action of calcium and bind strongly to sulfhydryl groups on proteins^4^.

In 2010, the United States Centers for Disease Control and Prevention (CDC) designated that the reference blood lead levels (BLLs) among adults and pregnant women should be lower than 10 and 5 μg/dL, respectively^9^,^10^. However, increasing evidence indicates that persistent low-level lead exposure has similarly devastating health consequences, especially for children and adolescents. Therefore, the consensus of many health organizations is that all forms of lead are toxic, and complete control and prevention of lead exposure is still far from being achieved^11^,^12^,^13^.

Although numerous occupational and public health measures have been employed to control lead exposure, lead poisoning still occurs today and is predominantly detected among people in developing countries and marginalized populations. According to statistics from the World Health Organization (WHO), lead poisoning accounts for approximately 0.6% of the global burden of disease^11^.

Currently, the only medical treatment for lead poisoning is the use of chelating drugs, such as meso-2,3-dimercaptosuccinic acid (DMSA)^14^. These drugs can save the lives of people with high BLLs; however, chelating treatment is typically implemented after long-term exposure and has been demonstrated to have limited value in reducing the sequelae of lead poisoning^15^,^16^. Therefore, controlling lead uptake, especially via the gastrointestinal tract, is an important strategy to prevent lead poisoning.

The metallothioneins (MTs) are a group of cysteine-rich proteins that are capable of binding heavy metal ions through the thiol groups of their cysteine residues. In mammals, two predominant MT isoforms, MT-I and MT-II, exist in all tissues^17^. Numerous studies have demonstrated that MTs are effective in binding lead and reducing lead-induced cytotoxicity^18^,^19^,^20^. However, it is impractical to scavenge lead ions from contaminated food and water via direct oral administration of MTs. The key obstacle is the production and ion-binding ability of recombinant human-derived MTs as well as various existing proteases in the digestive tract.

In previous studies, we expressed a soluble recombinant human MT-I fusion protein in *Escherichia coli* (*E. coli*) using glutathione S-transferase (GST) and small ubiquitin-related modifier protein (SUMO) tags. The purified recombinant GST-SUMO-MT bound metal ions and significantly protected multiple tissues against oxidative damage *in vivo*. More importantly, the recombinant host expressing GST-SUMO-MT had a higher survival rate than that of the control on the nutritional plates containing different concentrations of heavy metal ions^21^.

Compared to *E. coli*, the lactic acid bacteria (LAB) are more suitable for functioning as a drug delivery vector for the gastrointestinal tract^22^. *Lactococcus lactis* (*L. lactis*), a model LAB, is an anaerobic Gram-positive bacteria widely used in the food industry and considered a GRAS (generally regarded as safe) microorganism. *L. lactis* can survive in the gastrointestinal tract and does not produce endotoxic lipopolysaccharides (LPSs). In addition to having comprehensively studiedgenetic characteristics, *L. lactis* has been successfully developed as microbial factories to produce different heterologous proteins and therapeutic vectors to deliver proteins to the mucosal tissues via intranasal, oral, or genital mucosal surfaces^23^,^24^.

In this work, we constructed a constitutive expression vector to express the GST-SUMO-MT recombinant protein and transformed it into the *L. lactis* strain MG1363. The recombinant plasmid stability, the fermentation of the transformants and the ability of the MT-fusion protein to bind lead ions were subsequently assessed. Moreover, the protective ability of recombinant *L. lactis* against lead poisoning was evaluated via oral administration in a pubertal male rat model.

## Results

### Construction, expression and authenticity of recombinant GST-SUMO-MT

The deoxyribonucleic acid (DNA) fragments encoding the GST-SUMO-MT and GST-SUMO fusion proteins were inserted into pMG36e to obtain recombinant *L. lactis* expression plasmids pGSMT and pGS, respectively. The DNA sequencing results of the recombinant plasmids were identical to the predicted sequences. These plasmids were transformed into *L. lactis* strain MG1363 by electroporation. The reverse transcriptional polymerase chain reaction (RT-PCR) results indicated that the recombinant genes encoding GST-SUMO-MT and GST-SUMO were expressed (Supplementary Fig. S1A) in transformants. Glutathione sepharose 4FF affinity coulums chromatography was employed to purify the target proteins GST-SUMO-MT and GST-SUMO subsequently. The purity of these proteins was over 95%. Western blot analysis indicated that two proteins reacted positively with the anti-GST antibody (Supplementary Fig. S1B). Moreover, the authenticity of GST-SUMO-MT was confirmed by LC-MS/MS.and the coverage rate was 94% (Supplementary Fig. S2A,B).

### The lead-binding characteristics of recombinant GST-SUMO-MT

**Figure 1:**
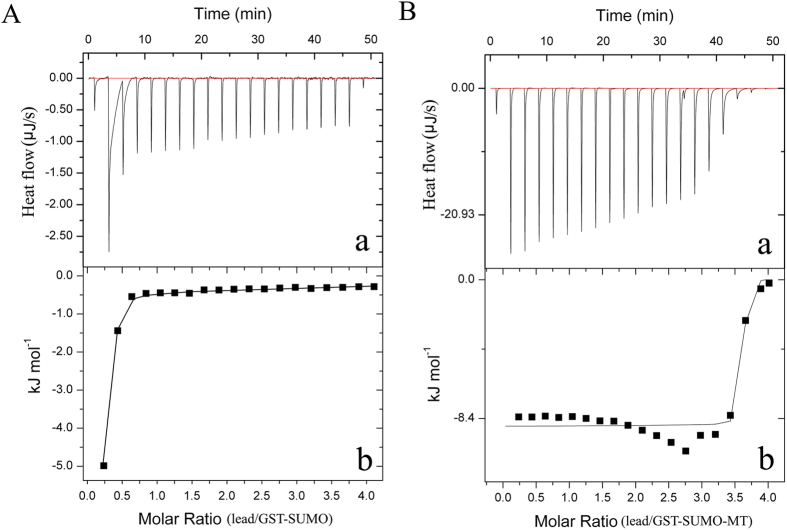
The isothermal titration calorimetry (ITC) analyses of the reaction of recombinant proteins binding lead ions. (**A**) ITC analyses of the reaction of GST-SUMO binding lead. (**a**) Represents the ITC raw data for 20 automatic injection of lead acetate solution (0.01 M) into the sample cell containing GST-SUMO solution (5×10^−5^ M). (**b**) Represents the plot and trendline of cumulative heat of injectant (corresponding to raw data of **a**) *vs* molar ratio of lead to GST-SUMO. (**B**) ITC analyses of the reaction of GST-SUMO-MT binding lead. (**a)** Represents the ITC raw data for 20 automatic injection of lead acetate solution (0.01 M) into the sample cell containing GST-SUMO-MT solution (5×10^−5^ M). (**b)** Represents the plot and trendline of cumulative heat of injectant (corresponding to raw data of **a**) *vs* molar ratio of lead to GST-SUMO-MT.

The heat signal is a universal property of binding reactions, so the isothermal titration calorimetry (ITC) was employed to measure the thermodynamic data of the reactions of recombinant proteins with lead ions. The data of the heatflow indicated that the mean binding number (n) of lead per GST-SUMO-MT and GST-SUMO molecule was 3.655 and 7.58×10^−6^, respectively (Fig. 1). Moreover, the changes of enthalpy (Δ*H*) and entropy (Δ*S*) during the reaction of GST-SUMO-MT binding each mole of lead were −8.2 kJ mol^−1^ and 301 J mol^−1^K^−1^. These results demonstrated that the reaction of GST-SUMO-MT binding lead is a spontaneous, exothermic and entropy-increasing process.

### The lead-binding functionality of recombinant *L. lactis* strains

**Figure 2:**
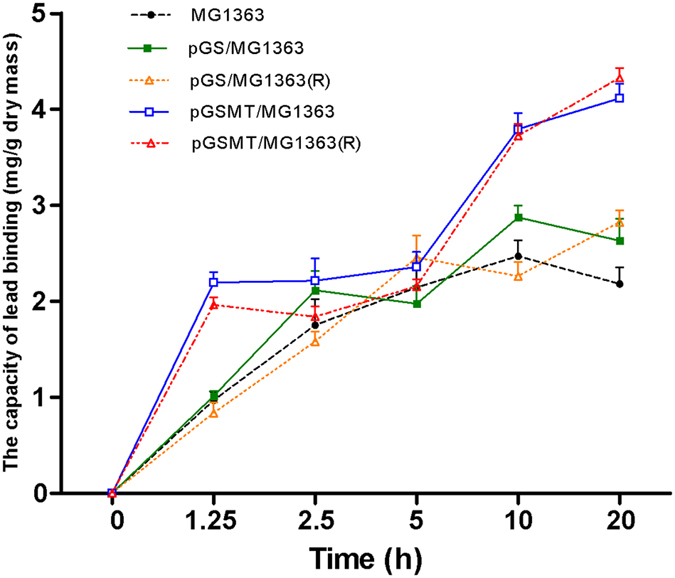
Lead-binding capacity analyses of MG1363, pGS/MG1363 and pGSMT/MG1363. The *L. lactis* strains were grown in GM17 medium with or without erythromycin until the OD_600_ reached 0.4. Then, lead acetate was added to a final concentration of 0.1 mM and the culture was incubated at 30 °C for up to 20 h. The cells were harvested and dried to constant weight. The mass of lead in these dried pellets was evaluated using ICP-OES (n = 3). The parameters are expressed as the means ± S.E.s. The curves represent the lead-accumulating tendency of the recombinant strains at different time points. The capital letter “R” represents the strains grown in the presence of erythromycin.

The Inductively Coupled Plasma-Optical Emission Spectrometry (ICP-OES) results showed that pGSMT/MG1363, pGS/MG1363 and the MG1363 were capable of binding lead ions, and the binding mass of lead increased gradually with the elongation of culture time (Fig. 2). The accumulation of lead in pGS/MG1363 and MG1363 increased rapidly during the 2.5 h culture and then remained in the plateau phase for the remaining culture period. The lead accumulation curve in pGSMT/MG1363 was significantly different. The accumulation was approximately 2.2 times higher compared with the other two strains during the initial 1.25 h of culture growth. After the 3.75 h plateau phase, the mass of lead accumulation had a further, remarkable increase. At the end of cultivation, the average lead concentration of pGSMT/MG1363, pGS/MG1363 and MG1363 was 4.11 ± 0.15, 2.63 ± 0.23 and 2.18 ± 0.17 mg/g of dry mass, respectively. Moreover, there were no significant differences in the lead-binding capacity between recombinant *L. lactis* strains cultured in medium with or without erythromycin.

### Growth of recombinant *L. lactis* in the lead-containing medium

**Figure 3:**
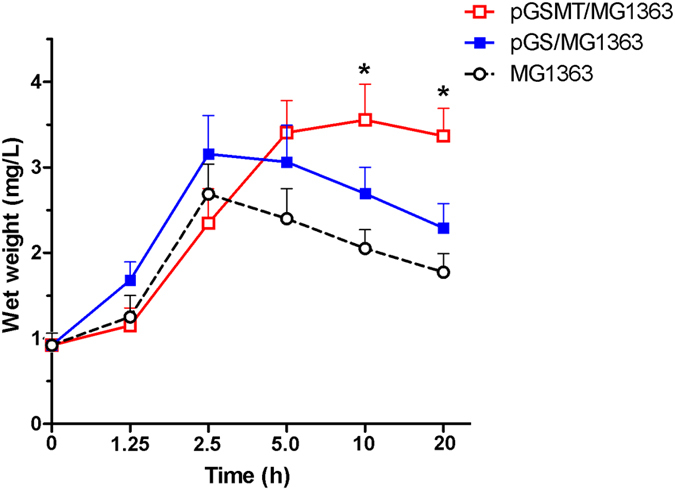
Growth tendency of *L. lactis* strains in the lead-containing medium. The *L. lactis* strains were inoculated into GM17 medium containing 0.1 mM lead for 20 h. The wet weight mass and optical density (OD_600_) of samples was measured at different time points (n = 4). The parameters are expressed as the means ± S.E.s. The asterisk indicates a significant difference at *p* < 0.05.

**Figure 4:**
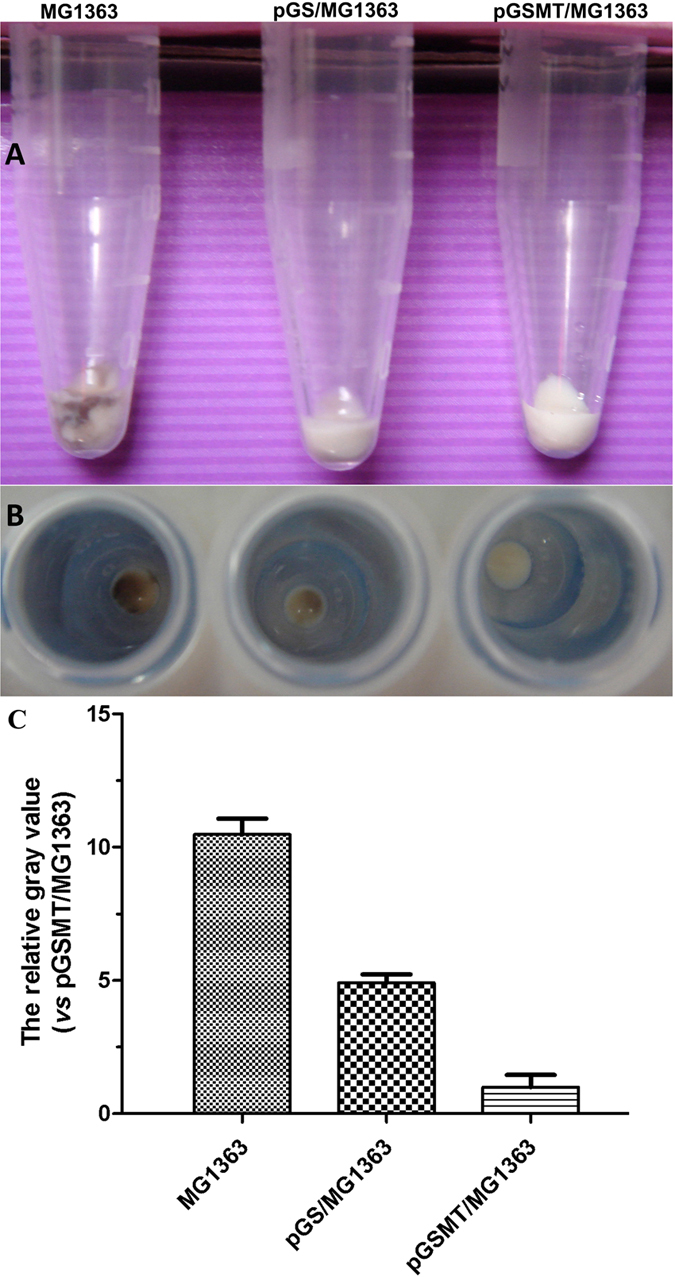
Morphological observations of recombinant *L. lactis* strains cultured with lead. The MG1363, pGS/MG1363 and pGSMT/MG1363 strains were cultured using GM17 medium containing a final concentration of 0.1 mM lead acetate at 30 °C. After a 20-h culture, the cells were collected and washed three times. The color observations of pellets at the end of culture ((**A**) side view; (**B**) top view); (**C**). The comparison diagram of average density scanned by Image J software. The parameters are expressed as the means ± S.E.s. (n = 3).

During the early culture period (~2.5 h), the growth rate of pGSMT/MG1363 was similar to that of pGS/MG1363 and MG1363. However, lead significantly inhibited the proliferation of pGS/MG1363 and MG1363 with increasing culture time (Fig. 3). The trend of curves indicated that the growth of these cells was inhibited significantly. By contrast, the proliferation and survival of pGSMT/MG1363 was not affected by lead. At the end of cultivation, the morphological observations showed that the surface colors of MG1363, pGS/MG1363 and pGSMT/MG1363 were deep brownish black, brownish black and grayish white, respectively (Fig. 4A,B). The scan data indicated that the surface color of pGSMT/MG1363 was 10.5 and 4.9 folds lighter than that of MG1363 and pGS/MG1363 (Fig. 4C). These findings suggested that the constitutively expressed GST-SUMO-MT maintained the cells survival via lead binding. The final average wet weight of pGSMT/MG1363 was 3.37 ± 0.22 mg/mL, which was 1.52 times higher that of pGS/MG1363 (*p* < 0.05).

### Stability of recombinant plasmid pGSMT

Because the fusion proteins were expressed by plasmid, it is very important that the plasmids and recombinant genes keep stable in *L. lactis* strains during culture period. The stability of the different plasmids in the recombinant strains was tested *in vitro* by subculture in M17-Bouillon medium with 0.5% glucose (GM17) in the presence or absence of erythromycin. After culture for a 30-day period with resistant plates, the recombinant pGSMT plasmid was remarkably stable in *L. lactis*. The DNA fragment encoding GST-SUMO-MT was positively detected in all clones by PCR. In the culture experiment using erythromycin-free GM17 medium continuously for 7 d, approximately 69.5 ± 3.5% clones survived on the GM17 medium containing erythromycin and the plasmids still harbored the GST-SUMO-MT fragment, as confirmed by colony PCR (Supplementary Fig. S3).

### Fermentation of pGSMT/MG1363

**Figure 5:**
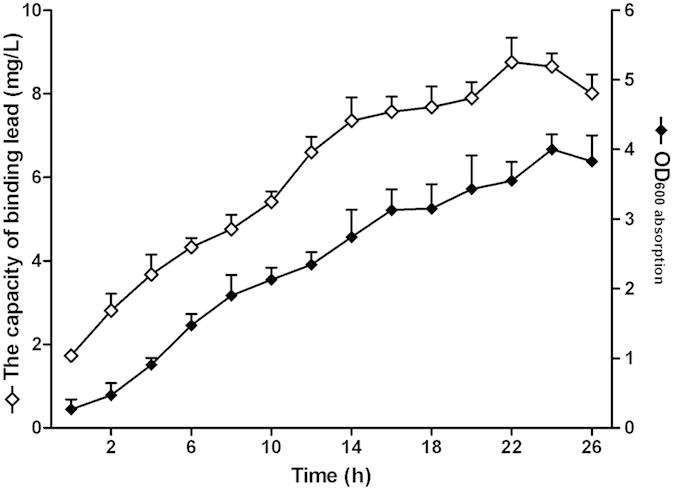
Fermentation of pGSMT/MG1363. The fermentation of pGSMT/MG1363 was performed in a 10-L fermenter under optimal conditions (30 °C, pH 6.0, 100 rpm agitation and 1.0 vvm aeration). Changes in the parameters including optical density and lead-binding capacity of pGSMT/MG1363 during the process of a 10-L fermentation were recorded by time course. The parameters are expressed as the means ± S.E.s. (n = 4).

Under the optimal growth conditions in a 10 L bioreactor, the highest cell density (OD_600_) was approximately 4.0, the dry biomass reached 2.03 ± 0.43 g/L and the average lead-binding capacity of pGSMT/MG1363 was 8.76 ± 0.58 mg/L (Fig. 5). To calculate the yield of recombinant *L. lactis*, we diluted the culture using sterile water, inoculated on GM17 plates and cultured at 30 °C for 20 h. The average clone number of recombinant pGSMT/MG1363 was 1.53 × 10^12^ CFU/L after fermentation.

### Lead concentration analyses of blood, feces and tissues

The lead-binding capacity and genetic characteristics of pGSMT/MG1363 were demonstrated by the aforementioned experiments. Next, we evaluated the preventative function of pGSMT/MG1363 on low-dose lead poisoning. After daily treatment for 6 weeks, neither the body weight nor the main tissue morphologies of the experimental animals significantly changed in comparison with the control group (Supplementary Fig. S4). However, the lead concentration of the main tissues, especially in the blood and kidney, significantly increased in the rats treated with 5 mg/kg lead daily (model group) (Table 1). Administration of pGSMT/MG1363 to the lead-treated rats provided significant prevention of lead accumulation in a dose-dependent manner. High-dose pGSMT/MG1363 (1 × 10^10^ CFU/d) decreased the lead concentrations in the blood, kidney and liver by approximately 2.8-, 1.5- and 2.5-fold, respectively, compared with the model group. The control, pGS/MG1363 (1 × 10^10^ CFU/d), improved only the lead concentration in the liver, compared with the model group (*p* < 0.05), and the preventive effects on other tissues were comparable with those of low-dose pGSMT/MG1363 (1 × 10^8^ CFU/d). DMSA effectively eliminated lead accumulation in the main tissues after continuous 7-d treatment. Interestingly, there was no significant difference in lead accumulation in the intestine between the model and experimental groups. In feces, the lead concentration of the DMSA-treated group was significantly lower than that of the model and *L. lactis*-treated groups, which most likely because over 90% DMSA is excreted in the urine^25^.

### Biochemical parameters of the serum

We subsequently assessed the lead-induced functional impairment of the main tissues based on serum biochemical parameters and prevention using recombinant *L. lactis*.

#### Liver

**Figure 6:**
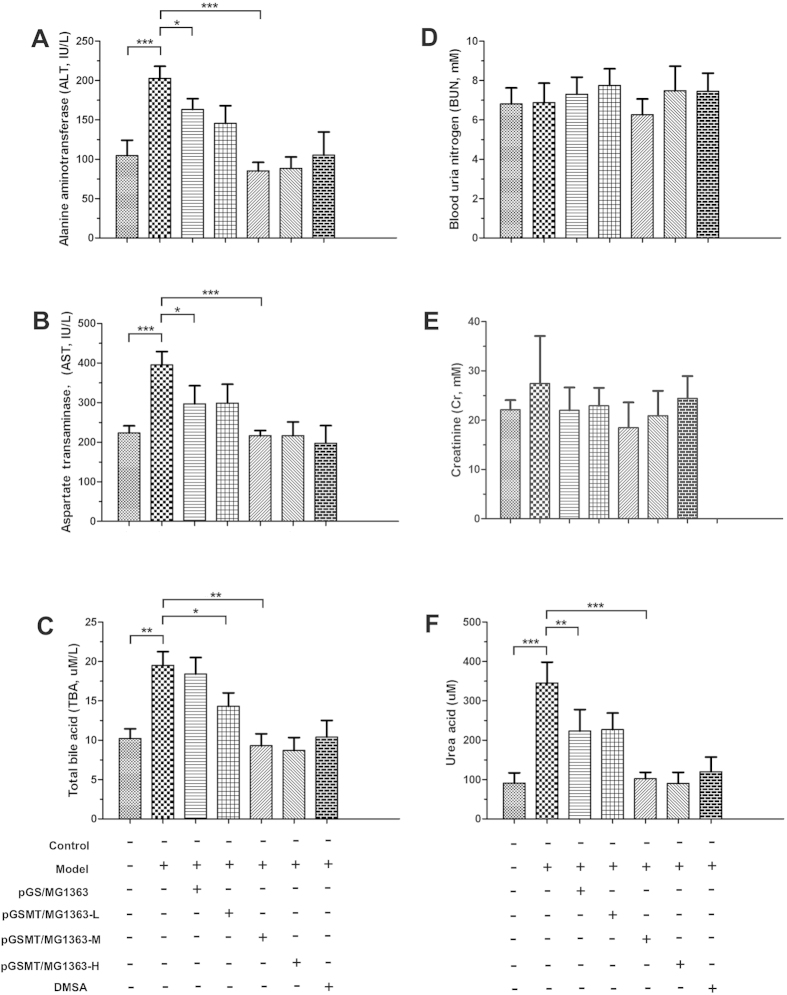
Preventative effects of pGSMT/MG1363 on hepatic (**A–C**) and renal (**D–F**) functions. The serum parameters are expressed as the means ± S.E.s. Control: rats without treatment; model: 5 mg/kg/d lead-treated rats; pGS/MG1363: lead-treated rats administered 1 × 10^10^ CFU/d; and pGSMT/MG1363-L, pGSMT/MG1363-M, and pGSMT/MG1363-H: lead-treated rats administered 1 × 10^8^, 1 × 10^9^, and 1 × 10^10^ CFU/d, respectively. n = 9. The asterisks, *, ** and ***, indicate significant differences at *p* < 0.05, *p* < 0.01 and *p* < 0.001, respectively.

After lead exposure for 6 weeks, the average alanine aminotransferase (ALT), aspartate aminotransferase (AST) and total bile acid (TBA) levels of the model group were 202.8 ± 15.2 IU/L, 395.4 ± 33.1 IU/L, and 19.5 ± 1.7 μmol/L, respectively, which were significantly higher than those of the control (*p* < 0.001). These results indicated that long-term exposure of low-dose lead resulted in impaired hepatic functions. Moreover, treatment with pGS/MG1363 could not effectively prevent the impairment of hepatic function by lead. The serum parameters of the rats treated with middle-dose and high-dose pGSMT/MG1363 were comparable with those of the control and DMSA-treated groups (Fig. 6A–C).

#### Kidney

Although the accumulation of lead in the kidney is higher than that of other soft tissues, there were no significant changes in the creatinine and urea levels between the control group and lead-treated rats with or without *L. lactis* (Fig. 6D,E). By contrast, lead elevated the serum urea acid 3.8 times higher than that of the control (Fig. 6F). This result suggested that lead impaired the urea acid disposing function of the kidney. The pGS/MG1363 strain suppressed the increase in urea acid concentration (decreased by 36% *vs* the model), but the urea acid was still significantly higher than the control (*p* < 0.01). Treatment with middle-dose pGSMT/MG1363 prevented the increase in urea acid concentration that was induced by lead and maintained normal levels.

#### Testis

**Figure 7:**
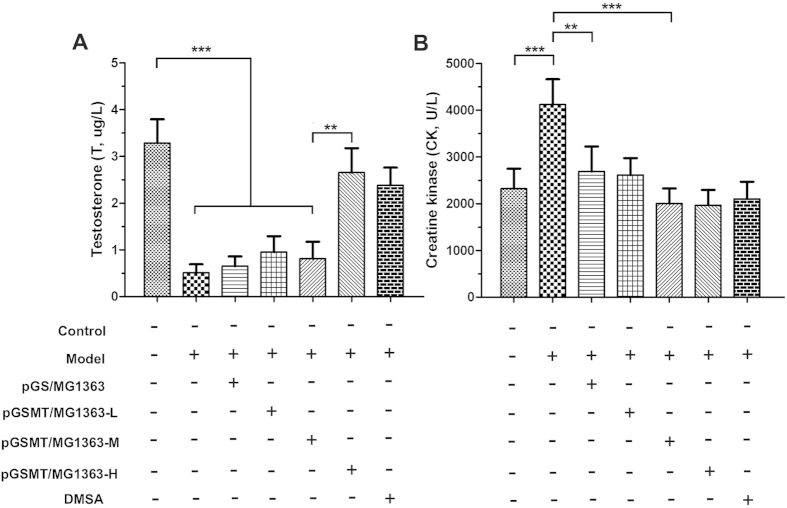
Creatine kinase and testosterone concentrations of rats treated with lead and recombinant *L. lactis* strains. The concentrations of creatine kinase and testosterone are expressed as the means ± S.E.s. Control: rats without treatment; model: 5 mg/kg/d lead-treated rats; pGS/MG1363: lead-treated rats administered 1 × 10^10^ CFU/d; and pGSMT/MG1363-L, pGSMT/MG1363-M, and pGSMT/MG1363-H: lead-treated rats administered 1 × 10^8^, 1 × 10^9^, and 1 × 10^10 ^CFU/d, respectively. n = 9. The asterisks, ** and ***, indicate significant differences at *p* < 0.01 and *p* < 0.001, respectively.

The Leydig cells of the testis are responsible for the synthesis and secretion of androgens, which are critical for the development and reproductive functions of males. The I^125^-based radioimmunoassay (RIA) results demonstrated that lead significantly suppressed testosterone synthesis and secretion (Fig. 7A). The average testosterone level of the lead-treated rats decreased by 84.5% compared with that of the control rats (*p* < 0.001). However, high-dose pGSMT/MG1363-treated and DMSA-treated rats maintained the normal serum concentration of testosterone. Moreover, the preventative effect of middle-dose pGSMT/MG1363 was significantly lower than high-dose pGSMT/MG1363 (*p* < 0.001), suggesting that the process of testosterone synthesis by Leydig cells was easily disturbed with low blood concentrations of lead.

#### Muscle or brain

Exposure to lead also induced elevated creatine kinase (CK) concentrations in the serum, which is seen in conditions that produce damage to the muscles or brain (Fig. 7B). The average levels of CK in the lead-treated and control groups were 4,122 ± 330 and 2,319 ± 223 U/L (*p* < 0.001), respectively. The middle- and high-dose pGSMT/MG1363 as well as DMSA were capable of inhibiting the elevation of CK.

#### Metal ions

The concentration of various metal ions in the serum was evaluated based on the metal-chelating characteristics of the MTs. Treatment with recombinant *L. lactis* for 6 weeks did not result in significant differences for the concentrations of calcium, magnesium, sodium, chlorine or potassium ion (Supplementary Fig. S5).

### Detection of endogenous MT expression in the intestine

**Figure 8:**
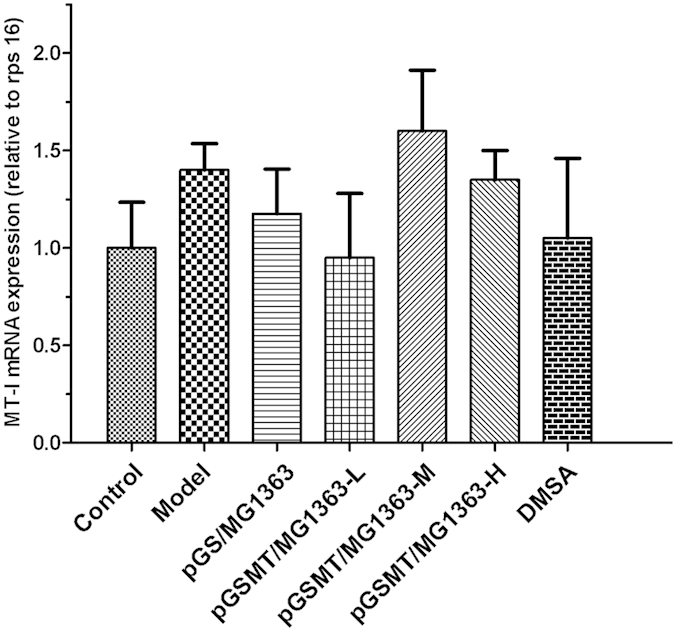
mRNA analysis of MT-I in the intestine after treatment with lead and recombinant *L. lactis* strains. The relative expression levels of *mt-I* detected by RT-PCR were compared with that of *rps 16* and expressed as the mean ± S.E.s. (n = 5).

MT is inducible by various stressors, but lead can not affect MT transcription. We performed RT-PCR of the rat MT messenger RNA (mRNA) to determine whether the lead prevention observed above is attributed to the administered pGSMT/MG1363 rather than induced MT expression by the intestinal cells. The RT-PCR result showed that there was no significant difference in the mRNA expression of MT between the control, lead-treated and experimental groups (Fig. 8). Combined with the aforementioned growth results of recombinant *L. lactis* in the lead-containing medium, this finding further confirmed that pGSMT/MG1363 was capable of preventing lead toxicity by binding lead using the GST-SUMO-MT fusion protein in the digestive tract.

## Discussion

Lead is one of the earliest metals discovered and has been widely applied by the human race. Although the toxicity of lead is well demonstrated and various measures have been adopted, chronic lead poisoning remains a problem of great importance for human health and development worldwide, especially for children and pregnant women. Recently, low-level exposure to lead (less than 10 μg/dl) has been demonstrated to adversely damage the nervous, immune, reproductive and cardiovascular systems. Therefore, reducing lead absorption is an effective prevention method for lead poisoning.

In this study, we constructed the recombinant *L. lactis* strain pGSMT/MG1363, which constitutively expresses the GST-SUMO-MT fusion protein that prevents lead from entering the body via the digestive tract. The expression level of the fusion protein was extremely low in *L. lactis*. It is well known that the intracellular production of heterogeneous proteins in *L. lactis* strains is far less than *E. coli* and yeast^26^,^27^. Moreover, the relatively large molecular weight (approximately 400 amino acids) of the fusion protein and because MTs often have toxic effects on their host strains, the expression and accumulation of the fusion protein may have been further restricted^28^,^29^. Efforts to improve expression need further investigations, but the addition of certain trace elements during pGSMT/MG1363 fermentation may help. Accroding to our existing experience, the expression level of GST-SUMO-MT and SUMO-MT in *E. coli* was improved significantly by adding cysteine and zinc sulfate during fermentation. Another option is to exchange the DNA fragment coding GST-SUMO-MT with host genes (non-essential multi-copies genes) by homologous recombination^30^. Although the authenticity of GST-SUMO-MT was confirmed using LC-MS/MS, 6% sequence did not be detected. The reason was likely that these sequences contained multiple lysines which were the digestive sites of trypsin. The length of fragments digested by trypsin was too short to meet the requirements of mass spectrometry detection.

Usually, a MT molecule could bind 7 lead ions. However, the mean lead number is 3.655 in GST-SUMO-MT treatment. The reason may be exist in that the GST-SUMO blocked the binding sites of the MT^31^. On the other hand, ITC results indicated that the reaction for GST-SUMO-MT binding lead is a spontaneous, energy releasing process. The value of enthalpy change was mainly attributed to the bonds formation of lead and sulfhydryl groups of MT. The increasing entropy value is partly attributed to the expansion of protein molecule volume after binding lead^32^.

The cell wall is a natural barrier for lead, since the main component of membrane including peptidoglycan together with teichoic acids and polysaccharides in lactic acid bacteria are involved in binding this metal^33^. This binding reaction is an uncontrol process, and the accumulation of lead increases and reaches maximum with the culture time increase. Lead could also enter bacterial cells through the uptake pathways for essential divalent metals^34^. After culture with GM17 medium containing lead for 20 h, the colors of the *L. lactis* pellets were remarkably different. We speculated that the brownish black colors of the MG1363and pGS/MG1363 pellets were formed by lead compounds, such as lead sulfide or lead oxide, that adsorbed on the surface of the hosts. By contrast, pGSMT/MG1363 maintained a grayish white color, which may be from lead chelation by MT and further reduction of lead accumulation on the bacterial surface.

The primary function of glutathione S-transferases is to detoxify xenobiotics; therefore, the lead-binding capacity and survival rate against lead of pGS/MG1363 was higher than that of MG1363. Differing from the lead-chelating mechanism of MT, the increase of lead accumulation in pGS/MG1363 (*vs* MG1363) may be related with the detoxified function of GST^35^. However, the enzymatic activity of GST could be inhibited rapidly, which would be accompanied by the increase in lead concentration and culture time^36^. The result shown the survival decrease of pGS/MG1363 but not pGSMT/MG1363, suggesting that the main toxicity prevention from lead is by MT but not GST-SUMO.

In the experiment of lead accumulation analysis, the curve in pGSMT/MG1363 was significantly different compared to the pGS/MG1363. It is likely that the rapidly increase of lead mass in initial 1.25 h of culture growth was due to the reaction of pre-existing GST-SUMO-MT binding lead. After consumption, the cells grew and expressed more recombinant protein which resulted in the emergence of the plateau phase. Following the increase of recombinant protein experssion, the lead accumulation mass of pGSMT/MG1363 had a further enhancement.

Leydig cells are the testicular cells responsible for androgen production. The entire postnatal developmental process of the Leydig cell population undergoes four phases: stem Leydig cells, progenitor Leydig cells (PLCs, 21 d postnatal), immature Leydig cells (ILCs, 35 d postnatal) and adult Leydig cells (ALCs, 90 d postnatal)^37^,^38^,^39^. During differentiation from ILCs to ALCs, almost all enzymes related to androgen synthesis were expressed at high levels^40^. After treating the pubertal male rats with low-dose lead for 6 weeks, the serum concentration of testosterone decreased significantly. This inhibitory effect on steroidogenesis may be relative to the simultaneously downregulated expression levels of steroidogenesis acute regulatory (StAR) protein, P450 side-chain cleavage enzyme (P450scc) and 3β-hydroxysteroid dehydrogenase (3β-HSD) that are mediated by lead^41^,^42^. Because the processes catalyzed by these enzymes are rate-limiting steps of androgen synthesis, a low concentration of lead could significantly inhibit the testosterone yield. Fortunately, the testosterone concentration could be restored to normal levels after removing the blood lead using DMSA.

In the present study, several serum parameters indicated that in addition to the testis, the liver was another tissue easily impaired by low-dose lead poisoning. This result is related to the metabolism of lead. After being absorbed in the body, lead reacts with thiol groups on peptides and proteins, inhibiting their normal activities. In the liver, hepatic glutathione attaches to lead and promotes its excretion in the feces. Once the hepatic glutathione is depleted, the liver is subject to the increasing toxic substances^13^,^43^.

Lead detoxification with pGSMT/MG1363 for 6 weeks did not result in any pathological effects of the tissues or depletion of essential nutrients including calcium and magnesium, indicating that this biomaterial is safe. Furthermore, GST-SUMO-MT bound zinc ions and thus could be applied as a delivery system for zinc supplementation during lead detoxification, based on the inherent binding characteristics of MT^21^,^44^.

In summary, we constructed a recombinant pGSMT plasmid and transformed it into *L. lactis* MG1363. The recombinant system bound intracellular lead ions by expressing GST-SUMO-MT, and the average lead-binding capacity was 4.11 ± 0.15 mg/g of dry mass. Daily oral administration of pGSMT/MG1363 reduced the blood lead level and further decreased the lead-induced impairment of liver, kidney and testis. Therefore, this study provides a simple biomaterial for preventing lead and other heavy metal ion poisoning in humans and mammals via the digestive tract.

## Materials and Methods

### Reagents

The pMG36e plasmid and the *L. lactis* MG1363 strain were obtained from Jinan Biopharmaceutical Research and Development Center (Guongzhou, China). *E. coli* strain JM109 was purchased from Promega Biotech Co., Ltd. (Madison, WI, USA). M17 broth medium was obtained from Thermo Fisher Scientific Inc. (Waltham, MA, USA). The Pyrobest DNA polymerase, restriction enzymes (*Xba* I and *Sph* I), DNA Marker, Plasmid Purification Kit, and DNA Fragment Purification Kit were obtained from Dalian Takara Corp. (Dalian, China). The synthetic gene and its primers were synthesized by GenScript Co., Ltd. (Nanjing, China). The Sprague-Dawley male rats (35 d postnatal) were purchased from the Experimental Animal Center of Guangdong Province (Guangzhou, China).

### Construction of the GST-SUMO-MT recombinant expression vector

The synthetic gene encoding the GST-SUMO-MT fusion protein was synthesized according to *L. lactis* preferred codon usage and cloned into pMG36e to create recombinant expression vector pGSMT. The accuracy of the inserted synthetic gene was confirmed by DNA sequencing. The recombinant plasmid was transformed into *L. lactis* MG1363 by electro-transformation using a Gene Pulser Xcell™ Electroporation Systems (Bio-Rad, Hercules, CA, USA) and is henceforth referred to as pGSMT/MG1363^45^. The transformed cells were selected on GM17 agarose plates containing 5 μg/ml erythromycin and incubated at 30 °C for 2–3 d. The control plasmid, pGS, and the transformants, referred to as pGS/MG1363, were also obtained by the methods described above. Illustrations of the fusion proteins are shown in Supplementary Fig. S6.

### Total RNA Extraction of *L. lactis* strains and the detection of recombinant genes

The single clone of MG1363, pGS/MG1363 and pGSMT/MG1363 was inoculated respectively in tube containing 5 mL of GM17 broth medium at 30 °C for 1 d. Cells of 1 mL were collected by centrifugation at 12,000 × g for 1 min at 4 °C. The pellets were lysed and the total RNA was extracted using HiPure Bacterial RNA Kit (Magen, Guangzhou, P.R. China). Total RNA of 1 μg was used as the template for cDNA synthesis primed with random hexamers (Bio-Rad, Hercules, CA, USA). The reaction mixture was incubated at 42 °C for 30 min followed by 5 min at 85 °C. The PCR conditions were as follows: one cycle at 94 °C for 3 min, followed by 35 cycles at 94 °C for 30 s, 55 °C for 30 s, and 72 °C for 1 min, with an additional extension at 72 °C for 7 min. Three DNA fragments (*mt, gst-sumo* and *16s rRNA*) were performed for each sample using different primers (Supplementary Table S1) and the PCR products were checked on 2% (w/t) agarose gel electrophoresis.

### Expressin and purification of GST-SUMO-MT in *Escherichia coli* (*E. coli*)

To investigate the lead-binding characteristics of GST-SUMO-MT and the control GST-SUMO, the DNA fragments encoding GST-SUMO-MT and GST-SUMO were synthesized by Invitrogen Company (Guangzhou, P.R. China) and cloned into the vector pET-20b to obtain the recombinant plasmids pET-GSMT and pET-GS, respectively. These two plasmids were transformed subsequently into *E. coli* Origami B (DE3) competent cells. The transformants were selceted using ampicillin resistance, and the accuracy of the inserted cDNA was confirmed using automated DNA sequencing by Shanghai Sangon Biological Engineering Technology and Services Co., Ltd (Shanghai, P.R. China).

Recombinants were inoculated in fresh 250-mL lysogeny broth (LB) medium and incubated at 37 °C on a shaker (250 rpm). When the optical density (OD_600_) of the culture reached 0.6–0.8, the temperature of incubation was shifted to 20 °C. Then the induction of recombinant proteins were performed by adding isopropyl thio-β-D-galactopyranoside (IPTG) and zinc sulfate solution to a final concentration of 1 mM and 0.5 mM respectively and incubating for 24 h.

After induction, the cells were harvested by centrifugation at 10,000 × g for 10 min at 4 °C and lysed by sonication. The purification of GST-SUMO-MT and GST-SUMO were performed according to the method described by Huang *et al*.^21^. Brifely, Glutathione Sepharose 4FF affinity coulums (GE Healthcare) were equilibrated using three column volumes of phosphate-buffered saline (PBS, pH 7.4). The lysis were loaded at a flow rate of approximately 1 mL/min. After washing with three column-volumes of PBS, the recombinant proteins were collected using the eluted buffer (0.05 M Tris-HCl buffer, 0.02 M glutathione, pH 8.0). The concentration of target proteins was quantified using BCA protein assay and the immunogenic activity were detected by western blotting.

### Evaluation of lead-binding functionality of fusion proteins

The lead-binding capabilities of recombinant GST-SUMO-MT and GST-SUMO were detected by using an isothermal titration calorimeter (ITC) accroding to the method described previously^32^. In brief, the purified GST-SUMO-MT and GST-SUMO diluted were dilutied into 0.01 M HClO_4_-NaAc buffer (pH 4.7) at a final concentration of 5×10^−5^ M. These proteins solutions, the lead acetate (0.01 M) and the control buffer of HClO4-NaAc were all deaerated with nitrogen for 15 min. For an isothermal titration, automated sequence of 20 injections, each of 2 μL, spaced at 150 s intervals were performed using a 100 μL injection syringe with stirring at 750 rpm. All the titration experiments were carried out at 298.15 K and repeated three times. Thermodynamic data were collected every 5 s by the software of MicroCal iTC200.

### Expressin and purification of GST-SUMO-MT in pGSMT/MG1363

The single clone of recombinant pGSMT/MG1363 was grown in 50 mL of GM17 medium at 30 °C without shaking for 24 h. The culture was then transferred into a 1-L GM17 medium containing 0.5 mM zinc sulfate and cultured at 30 °C without shaking for 48 h. The cells were collected by centrifugation at 12,000 × g for 20 min at 4 °C and the pellets (approximate 1 g wet weight) were resuspended in 10 mL PBS lysed by sonication. The recombinant protein in the supernatant was pooled using the purified method described above. The volume of pools containing GST-SUMO-MT was further concentrated by using ultrafilter with 3 kDa cutoff membrane at 4 °C. The specific band on the SDS-PAGE gel was digested with trypsin and the authenticity of recombinant GST-SUMO-MT was confirmed using liquid chromatography and tandem mass spectrometry (LC-MS/MS) analysis by National Center of Biomedical Analysis (Beijing, P.R. China).

### Evaluation of lead-binding functionality of recombinant strains

Transformants were inoculated in tubes containing 5 mL of GM17 broth medium. These tubes were placed in a wide neck glass flask, which was sealed and the oxygen inside was consumed using a lighted candle. Then, the flask with the tubes was cultured at 30 °C without shaking. After culturing for 20 h, the sample was transferred into 95 mL of fresh GM17 broth medium (with or without 5 μg/mL erythromycin) until the OD_600_ reached 0.4. Then, a lead acetate solution was added at a final concentration of 0.1 mM, and the culture was incubated at 30 °C for up to 20 h in same anaerobic environment. The cells were harvested by centrifugation at 12,000 × g for 5 min at 4 °C. The pellets were washed three times with 5 mL of ice-cold GM17 medium and dried at 105 °C for 24 h to constant weight. These dried materials were dissolved by a 65% nitric acid solution (v/v) at a final concentration of 10 mg/mL. A 1-mL volume of this solution was diluted with 4 mL of ultra-pure water and the concentration of lead was analyzed by ICP-OES (Optima 2000 DV, PerkinElmer Inc., USA). The lead detection limit of ICP-OES is 25 μg/g, and this experiment was repeated in triplicate.

### Growth of recombinant *L. lactis* in lead-containing medium

To evaluate the growth rate of recombinant *L. lactis* in the lead-containing GM17 medium, pGSMT/MG1363, pGS/MG1363 and the MG1363 negative control (vector free) were cultured in 5 mL of GM17 broth at 30 °C under anaerobicconditions. After 20 h of incubation, the broth was transferred to GM17 broth medium containing 0.1 mM lead at a ratio of 5% and continually cultured for approximately 20 h. The wet weight mass and optical density (OD_600_) of the samples were measured at different time points. The morphology of cells was quantified using Image J software (Bio-Rad Laboratories, CA, USA) and normalized to pGSMT/MG1363.

### Fermentation of recombinant pGSMT/MG1363

Fermentation of the recombinant *L. lactis* pGSMT/MG1363 was performed in a 10 L jar fermenter (BTF-10SIP, Biotop Inc., Chinese Taipei), with a 7-L working volume of GM17 medium. The temperature, pH, agitation and aeration rate was maintained at 30 °C, 6.0, 100 revolutions per minute(rpm) and 1.0 air volume/culture volume/min(vvm), respectively. Cell density was evaluated by both optical density (OD_600_) and dry cell weight (n = 4). According to the method described above, lead-binding capacity analyses were performed on the samples harvested from different time points. After 26 h of fermentation, the cells were harvested by centrifugation at 4,000 rpm for 30 min at 4 °C and frozen at −20 °C for future experiments.

### Stability analyses of the recombinant pGSMT plasmid

Three clones harboring pGSMT were incubated in 5 mL of GM17 medium containing 5 μg/mL erythromycin at 30 °C without shaking. After incubation for 24 h, the cells were collected, washed two times and resuspended. Subsequently, the cultures were transferred into 5 mL of GM17 medium without erythromycin at a 1:20 ratio and cultured for 12 h at 30 °C. This growth cycle pattern in antibiotic-free GM17 medium was performed continuously for 7 d. To determine the stability of recombinant pGSMT, 10 μL of the full-grown culture was collected and diluted 10^5^-fold using sterilized water. The diluted culture (100 μL) was plated on a GM17 plate lacking erythromycin. After culturing for 16 h at 30 °C, 100 clones were randomly selected and inoculated onto resistant GM17 plates (containing erythromycin) and GM17 plates (without erythromycin) for 20 h at 30 °C. The emerging clones from these plates were scored and the percentage of plasmid retention was calculated with the following equation: % clones with plasmid = clones with erythromycin/clones without erythromycin × 100. In total, 10 resistant clones were selected and the gene coding the GST-SUMO-MT fusion protein was confirmed using the colony PCR method.

The stability of the recombinant pGSMT plasmid in the strains was also tested using GM17 plates containing 5 μg/mL erythromycin. A total of 100 clones were inoculated and subcultured daily on fresh resistant plates over a 30-d period. The recombinant gene encoding the GST-SUMO-MT fusion protein was detected by colony PCR.

### Animals and treatment

Sixty-three pubertal male Sprague-Dawley rats (35 d postnatal, approximately 180 g) were purchased from the Experimental Animal Center of Guangdong Province and randomly divided into seven groups (n = 9 per group): control, model, pGS/MG1363 treatment, pGSMT/MG1363 treatment (recombinant *L. lactis* at three dose levels) and DMSA. All *L. lactis* cultures were freshly prepared every day. The model group was given a daily oral administration of lead acetate solution (5 mg/kg body weight). The control group was given a daily oral administration of the same volume of saline. The three pGSMT/MG1363 dose levels were high (1 × 10^10^ CFU/d), medium (1 × 10^9^ CFU/d) and low (1 × 10^8^ CFU/d) and indicated by pGSMT-H, pGSMT-M and pGSMT-L, respectively. The pGS/MG1363 group, as a negative control, was given pGS/MG1363 daily at a 1 × 10^10^ CFU dose level. For lead-protection experiments, male pGSMT/MG1363 and pGS/MG1363 rats were orally administered the recombinant *L. lactis* daily and also a lead acetate solution (5 mg/kg body weight) 4 h after *L. lactis* treatment. This experiment was performed for 6 weeks. For the DMSA treatment group, the rats were treated daily with a lead acetate solution (5 mg/kg body weight) for 6 weeks and then daily oral administration of DMSA (32 mg/kg body weight) for 7 d. The animals were killed using carbon dioxide suffocation. The blood and tissues were collected quickly and stored at −80 °C for the subsequent determination of lead and histological examination. All animal experiments were performed in accordance with the National Institutes of Health Guidelines for Experimental Animals and were approved by the Animal Care and Use Committee at Jinan University.

### Determination of lead concentration in whole blood and other tissues

The accumulation of lead in tissues was determined by atomic absorption spectrometric analysis as described by Kosik-Bogacka^46^. Briefly, 2 mL of blood or 0.5 g of other soft tissues were digested in 25-ml volumetric flasks with 4 mL of 16 N HNO_3_ and 1 mL of 11.6 N HClO_4_. Sample heating was commenced at 60 °C for 2 h, and then increased 20 °C every 2 h to 160 °C. When cooling down, 5 mL of ultra-pure water were added to the samples and heated at 160 °C to allow the solution to evaporate to 2–3 mL. After cooling, the solution was diluted with ultra-pure water to 25 mL and used for the detection of lead concentration with a graphite furnace atomic absorption spectrometer (GFAAS, Shimadzu AA-6300, Japan). A 283.3 nm wavelength was used for monitoring and the operating conditions of the GFAAS were DRY at 150 °C for 20 s and 250 °C for 10 s; CHAR at 800 °C for 23 s; and ATOMIZE at 2400 °C for 2 s and 2500 °C for 2 s. The sensitivity of this assay is 0.5 μg/g and the results from at least four samples in each group were averaged for the statistical analysis.

### Biochemical assays

The hematology and serum biochemistry parameters were measured using a fully automated Hematology analyzer. The serum testosterone concentrations were measured using an I^125^-based radioimmunoassay (RIA) as described previously^47^. Briefly, the standards, controls, and serum samples (50 μL, in duplicate) were dispensed into numbered tubes. Subsequently, 100 μL of an I^125^-labeled testosterone tracer and 100 μL of the primary antibody were added to the appropriate tubes. The tubes were shaken for 10 s and incubated in a water bath for 1 h at 37 °C. Then, 500 μL of the secondary antibody was added to all tubes and incubated for 15 min at room temperature. The tubes were then centrifuged at 1,800 × g and 4 °C for 15 min. The supernatants were decanted, and the radioactivity in the precipitate was counted for 1 min. The sensitivity of this assay system was 0.01 μg/L. The intra-assay and inter-assay variations were less than 10 and 15%, respectively. The results from all samples (n = 9) were averaged for the statistical analysis.

### Quantitative analysis of endogenous expression of intestinal MT

After administration of lead or the recombinant *L. lactis* strains for 6 h, the rats were sacrificed, and the intestine was isolated. For RT-PCR, the total RNA from the intestine was extracted and used as templates for cDNA synthesis. All PCRs were performed using a Bio-Rad CFX Connect Real-Time system (Bio-Rad Laboratories, CA, USA), and the data were collected using Bio-Rad CFX Manager software (version 2.0). The primers used to amplify the mRNA of *mt-I* (rat) and *rps16* are listed in Supplementary Table S1 and synthesized by the Beijing Genomic Institute (BGI, Shenzhen, China). The relative mRNA levels of the targeted genes were normalized to that of ribosomal protein S16 (*rps16*). The parameters for RT-PCR were one cycle at 95 °C for 30 s and then 40 amplification cycles at 95 °C for 15 s and 60 °C for 10 s.

### Statistical analysis

The data were analyzed by one-way ANOVA followed by Tukey’s ad hoc multiple comparison tests. All data are expressed as the means ± the S.E.s (standard errors). The differences were regarded as significant at *p* < 0.05, *p* < 0.01 and *p* < 0.001.

## Additional Information

**How to cite this article**: Xiao, X. *et al*. Prevention of gastrointestinal lead poisoning using recombinant *Lactococcus lactis* expressing human metallothionein-I fusion protein. *Sci. Rep.* **6**, 23716; doi: 10.1038/srep23716 (2016).

## Supplementary Material

Supplementary Information

## Figures and Tables

**Table 1:** Lead concentration in blood, kidney, brain, liver, intestineand feces.

Groups	Blood (μg/dl)	Kidneys (μg/g)	Brains (μg/g)	Liver (μg/g)	Intestine (μg/g)	Feces (μg/g)
Control	0.242 ± 0.156^a^	0.009 ± 0.006^a^	0.047 ± 0.015^a^	0.023 ± 0.021^a^	0.457 ± 0.189^a^	2.62 ± 1.25^a^
Model	10.476 ± 1.109^b^	2.732 ± 0.611^b^	0.121 ± 0.030^a^	0.649 ± 0.080^b^	1.630 ± 0.621^b^	570.52 ± 163.22^b^
pGS/MG1363	9.466 ± 0.843^b^	2.635 ± 0.518^b^	0.098 ± 0.027^a^	0.468 ± 0.024^c^	1.661 ± 0.347^b^	562.88 ± 243.03^b^
pGSMT/MG1363-L	8.700 ± 0.547^b^	2.648 ± 0.462^b^	0.079 ± 0.048^a^	0.513 ± 0.092^b^	2.468 ± 0.925^b^	519.45 ± 174.39^b^
pGSMT/MG1363-M	5.634 ± 0.455^c^	1.831 ± 0.191^c^	0.070 ± 0.039^a^	0.319 ± 0.029^d^	2.089 ± 0.654^b^	738.63 ± 138.98^b^
pGSMT/MG1363-H	3.724 ± 0.524^d^	1.787 ± 0.533^c^	0.045 ± 0.024^a^	0.260 ± 0.041d,e	1.935 ± 0.645^b^	620.61 ± 207.36^b^
DMSA	1.429 ± 0.517^e^	1.420 ± 0.214^c^	0.034 ± 0.018^a^	0.190 ± 0.069^e^	2.219 ± 0.765^b^	77.39 ± 31.75^c^

Values are mean ± S.E.s. (n = 9). ^a–e^Values with matching symbol notation within the same column are not statistically significant at the 5% level of probability.
