# Supplementary material for: Prevention of gastrointestinal lead poisoning using recombinant Lactococcus lactis expressing human metallothionein-I fusion protein
Source: Sci Rep. 2016 Apr 5;6:23716. doi: 10.1038/srep23716 (PMC4820694; doi:10.1038/srep23716)
Supplement: Supplementary Information [file srep23716-s1.doc]

**Supplementary material:**

**Scientific Reports**

**Title:**

Prevention of gastrointestinal lead poisoning using recombinant *Lactococcus lactis* expressing human metallothionein-I fusion protein

**Authors:**

Xue Xiao, Changbin Zhang, Dajun Liu, Weibin Bai, Qihao Zhang, Qi Xiang, Yadong Huang, Zhijian Su

**Corresponding author:**

**Zhijian Su**

Guangdong Provincial Key Laboratory of Bioengineering Medicine, Jinan University, Guangzhou 510632, China.

E-mail: [tjnuszj@jnu.edu.cn](mailto:tjnuszj@jnu.edu.cn)

**Yadong Huang**

National Engineering Research Center of Genetic Medicine, Jinan University, Guangzhou 510632, China.

E-mail: [tydhuang@jnu.edu.cn](mailto:tydhuang@jnu.edu.cn)

**Supplementary figure legends:**

**Suppl. Fig. S1. The reverse transcriptional PCR (A) and western blot (B) analyses of recombinant strains.**

1. 1. MG1363; 2.pGS/MG1363; 3. pGSMT/MG1363.
2. 1. MG1363; 2. pGSMT/MG1363; 3. pGS/MG1363.

**Suppl. Fig. S2. Peptides identification of the GST-SUMO-MT by liquid chromatography-tandem mass spectrometry (LC-MS/MS).**

1. The deduced amino sequences of the peptides identified on LC-MS/MS.
2. The amino acid sequence of GST-SUMO-MT from N to C terminal was numbered using an Arabic numeral on the left of each line. The letters marked in red represented that the deduced amino sequences of the peptides based on LC-MS/MS matched well with predicted GST-SUMO-MT. GST sequence was underlined with a dotted line, SUMO sequence was underlined with a solid line and MT sequence was indicated by solid and dotted line, respectively.

**Suppl. Fig. S3. Agarose gel electrophoresis of the DNA fragment encoding GST-SUMO-MT, as detected by direct colony PCR.**

Lane M, DNA marker; lane P, positive control (pGSMT plasmid as the template); lane N, negative control (without template); and lanes 1 to 9, the positive transformants.

**Suppl. Fig. S4. Body weight of lead-treated rats with or without the recombinant *L. lactis* strains (n=9).**

**Suppl. Fig. S5. Metal ions concentrations in the serum of rats treated with lead and recombinant L. lactis strains.**

**Suppl. Fig. S6. Schematic illustration of the fusion proteins.**

*Xba* I and *Sph* I indicate the names of restriction endonucleases used for recombinant DNA cloning.

**Suppl. Table S1. PCR primers for amplification of different genes.**


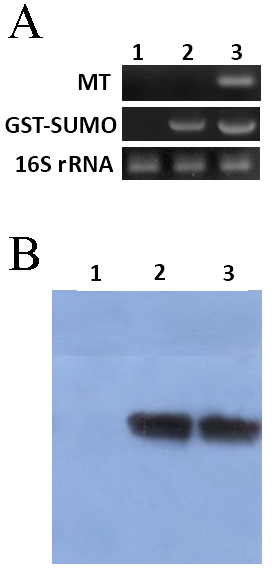


**Suppl. Fig. S1. The reverse transcriptional PCR (A) and western blot (B) analyses of recombinant strains.**


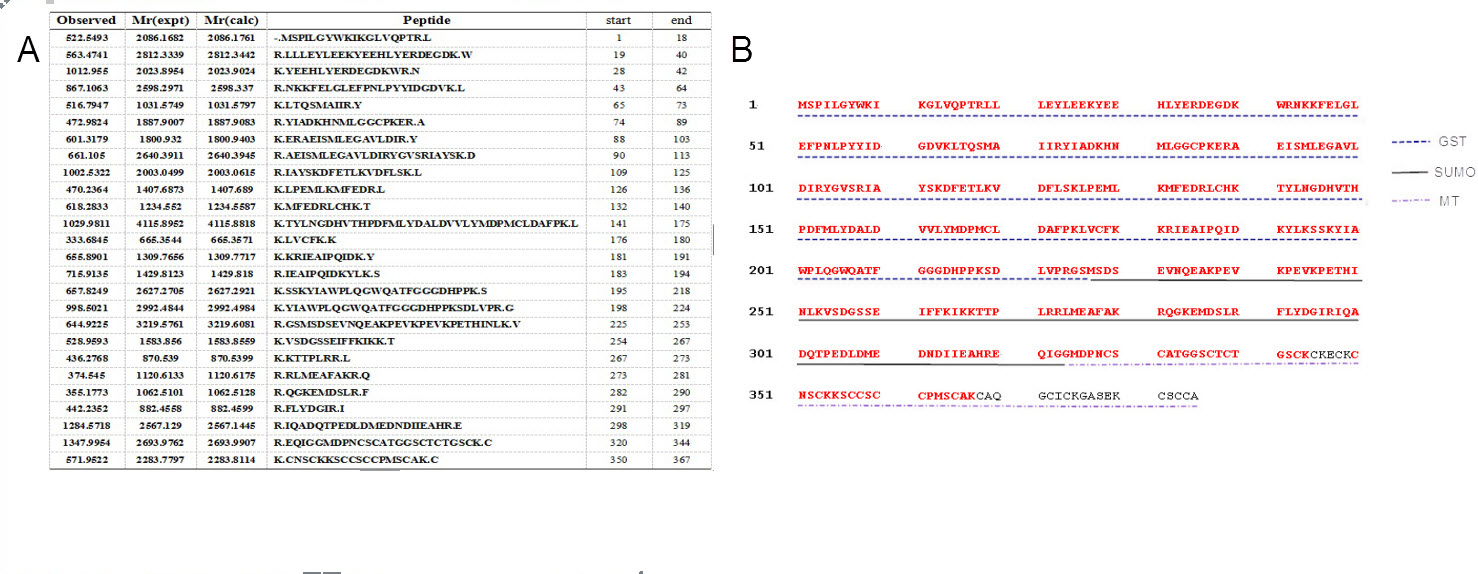


**Suppl. Fig. S2. Peptides identification of the GST-SUMO-MT by liquid chromatography-tandem mass spectrometry (LC-MS/MS).**


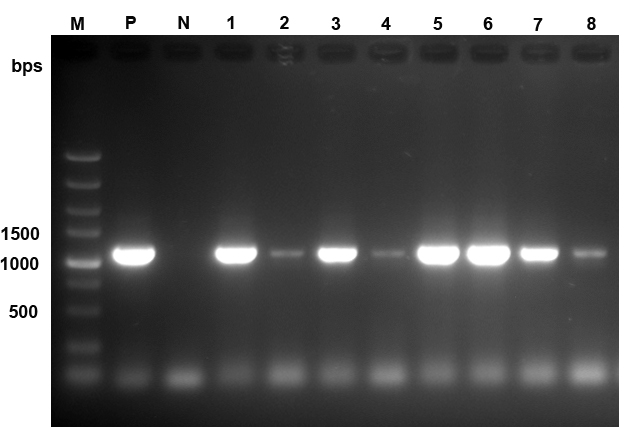


**Suppl. Fig. S3. Agarose gel electrophoresis of the DNA fragment encoding GST-SUMO-MT, as detected by direct colony PCR.**


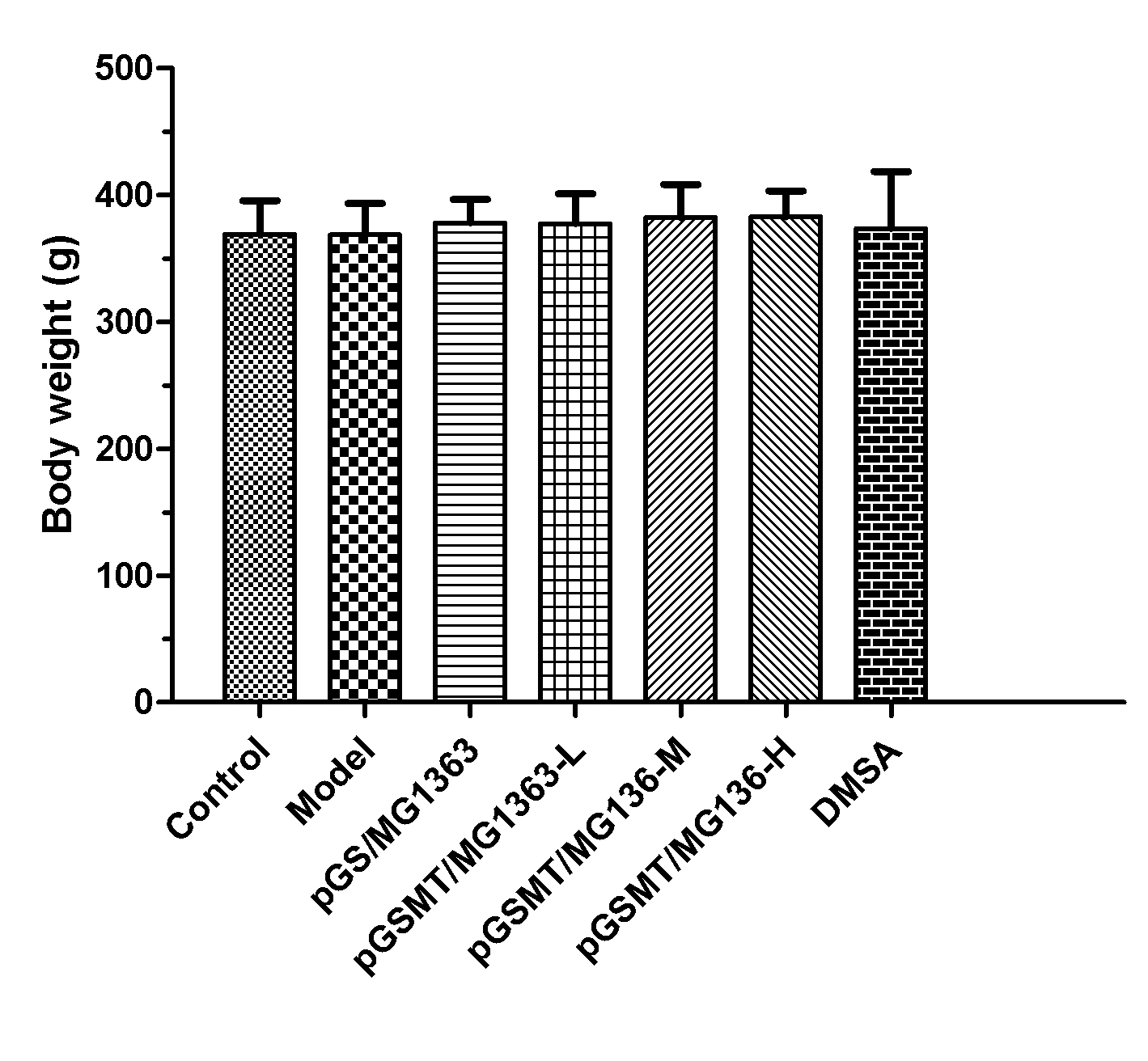


**Suppl. Fig. S4. Body weight of lead-treated rats with or without the recombinant *L. lactis* strains (n=9).**


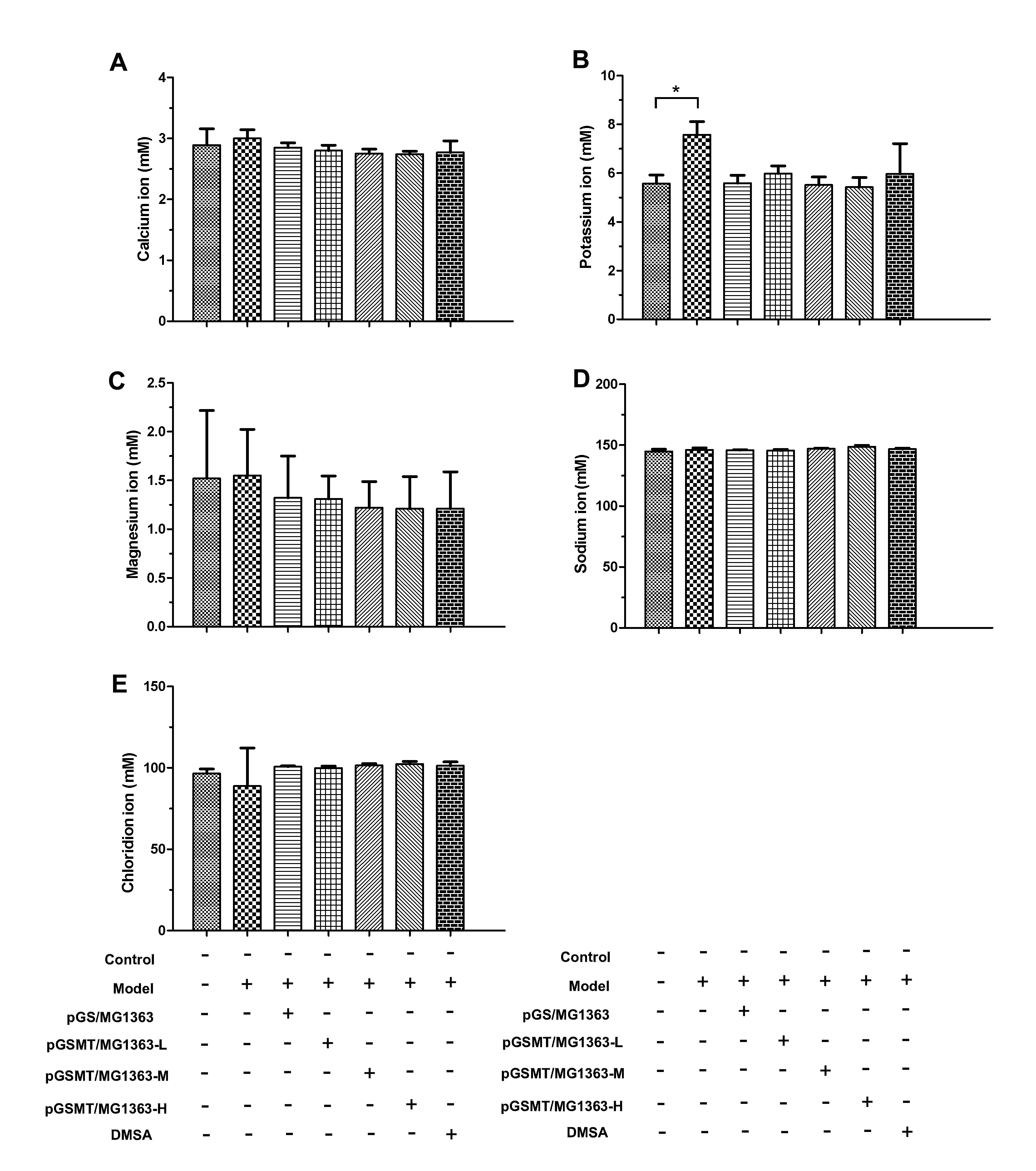


**Suppl. Fig. S5. Metal ions concentrations in the serum of rats treated with lead and recombinant L. lactis strains.**


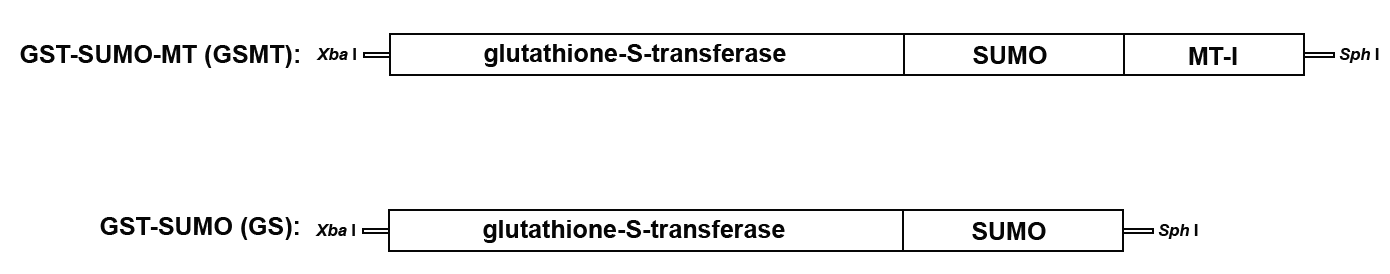


**Suppl. Fig. S6. Schematic illustration of the fusion proteins.**

**Suppl. Table 1. PCR primers for amplification of different genes.**

| **Gene** | **Sequence(5’—3’)** | **Product (bps)** | **GenBank Accession No.** |
| --- | --- | --- | --- |
| *mt-1* (rat) | Sense: TGTGCCTGAAGTGACGAACAG  Anti-sense: TCACATGCTCGGTAGAAAACG | 101 | NP_620181 |
| *rps16* | Sense: AAGTCTTCGGACGCAAGAAA  Anti-sense: TGCCCAGAAGCAGAACAG | 130 | NM_001169146 |
| *gst-sumo-mt* | Sense: AGCTCTAGAATGTCACCAATTTTAGGATATTG  Anti-sense: ACATGCATGCTTAAGCACAACATGAACA | 1186 | KP858143 |
| *16s rRNA* | Sense: TTTGAGCGGGGGACAACATT  Anti-sense: TGCCGAAGATTCCCTACTGC | 244 | AM406671 |
| *mt* (human) | Sense:ATGGATCCAAATTGTTCATGTGCTAC  Anti-sense:TTAAGCACAACATGAACATTTTTCTG | 186 | KP858143 |
| *gst-sumo* | Sense: AGCTCTAGAATGTCACCAATTTTAGGATATTG  Anti-sense: TCCTCCAATTTGTTCACGATGAGC | 990 | KP858143 |

The restriction enzyme recognition sites used for cloning were *Xba* I (sense) and *Sph* I (antisense) and the sites are indicated by boxes.
